# Factors influencing health information system acceptance: a cross-sectional study from a low-middle-income country

**DOI:** 10.3389/frhs.2024.1458096

**Published:** 2024-09-30

**Authors:** Raya Sawalha, Fayez Ahmad, Hamzeh Al Zabadi, Abdulsalam Khayyat, Samar Thabet Jallad, Tareq Amro, Rami Zagha

**Affiliations:** ^1^ Department of Public Health, Faculty of Medicine and Health Sciences, An-Najah National University, Nablus, Palestine; ^2^ Public Health Management Program, Faculty of Graduate Studies, An-Najah National University, Nablus, Palestine; ^3^ Nursing Department, Health Professions Faculty, Al-Quds University, Jerusalem, Palestine; ^4^ Mathematics Department, Faculty of Science and Technology, Hebron University, Hebron, Palestine; ^5^ Microbiology, Immunology, and Pathology Department, Faculty of Medicine and Health Sciences, An-Najah National University, Nablus, Palestine

**Keywords:** health information system (HIS), Avicenna, healthcare providers, acceptance, Palestine

## Abstract

**Background:**

The Avicenna unified Health Information System (HIS) was implemented by the Palestinian Ministry of Health in 2010 across government hospitals. Despite its potential, the acceptance of Avicenna HIS by healthcare providers remains unclear after 14 years of application. Understanding the factors that influence healthcare provider acceptance is essential for optimizing the system's success. We investigated factors affecting acceptance of Avicenna HIS among healthcare providers in Palestinian healthcare institutions, focusing on perceived usefulness, ease of use, human factors, technological factors, and organizational support.

**Materials and methods:**

A cross-sectional study was conducted at the Palestine Medical Complex (PMC) in Ramallah, West Bank, where the Avicenna HIS has been fully implemented since 2010. A systematic random sampling was used to select participants, resulting in 300 completed questionnaires. The study utilized a self-administered questionnaire adapted from a structured tool based on the Technology Acceptance Model (TAM). The questionnaire was validated through expert review and pilot testing, achieving a Cronbach's alpha of 0.86. Each selected healthcare provider was contacted face-to-face, and written informed consent was obtained before administering the questionnaire.

**Results:**

A total of 300 questionnaires were completed and returned. The study sample included 178 males (59.3%) and 122 females (40.7%). The majority of participants was aged 20–39 years (270 participants, 90%) and held a bachelor's degree (250 participants, 83.3%). Nurses comprised the largest professional group (153 participants, 51.0%). High levels of perceived usefulness and ease of use were reported, both with mean scores of 4.511 (S.D. = 0.295). Technological factors had a mean score of 4.004 (S.D. = 0.228), while organizational factors scored 2.858 (S.D. = 0.304). Overall acceptance of the HIS was moderately high, with a mean score of 4.218 (S.D. = 0.387). Significant differences in perceived usefulness and ease of use were noted based on gender, age, and experience.

**Conclusion:**

This study concludes that both technological and human factors significantly influence the acceptance of HIS among healthcare providers in Palestine. To improve HIS adoption, it is recommended to enhance system functionality, ensure reliable data quality, and provide comprehensive training programs for healthcare providers.

## Background

Technological advancements have significantly transformed various sectors, including healthcare. Information technology (IT) plays a pivotal role in shaping healthcare policies and decisions, ultimately enhancing the quality of care provided. A key determinant of an effective healthcare system is its ability to deliver integrated, high-quality care to the population (1). Health Information Systems (HIS) have emerged as vital tools for achieving this goal by facilitating data collection, documentation, retrieval, and information sharing among healthcare providers (2). These systems have notably improved the efficiency and quality of healthcare services in hospitals and other healthcare institutions (3).

HIS are instrumental in establishing comprehensive and integrated healthcare systems that cater to diverse health needs and contribute to cost reduction in healthcare delivery, which is a major concern for policymakers (4). However, the successful implementation of HIS depends significantly on user acceptance, particularly among healthcare providers. This acceptance varies due to numerous factors, including human, organizational, and technological elements (5). Understanding these factors is essential for optimizing the utilization of HIS and ensuring their positive impact on clinical decision-making and quality of care (6, 7).

In Palestine, the Ministry of Health (MOH) has prioritized the delivery of high-quality healthcare to improve health outcomes for its population. Since 2010, the Avicenna HIS has been gradually implemented across Palestinian government hospitals to enhance service quality, efficiency, and effectiveness (8, 9). Despite these efforts, healthcare provider (HCP) acceptance of the Avicenna HIS has been inconsistent, potentially hindering the full realization of its benefits (10).

The adoption of HIS can faces various challenges, such as interoperability issues, privacy concerns, and technological limitations, which can impede successful implementation (11, 12). Human factors, including healthcare providers' beliefs, perceptions, skills, compatibility with existing processes, adherence to information security standards, self-efficacy, and social influence, are crucial for HIS adoption (1, 13). Technological factors, like system quality and information accuracy, are also essential determinants of HIS success (4, 14). Moreover, organizational factors, such as effective management practices, comprehensive user training, and committed support staff, significantly influence the acceptability and successful implementation of HIS (15).

This study aims to investigate the factors influencing the acceptance of the Avicenna HIS among healthcare providers in Palestinian healthcare institutions. By focusing on human, organizational, and technological aspects, this research seeks to understand how these factors affect the acceptance and utilization of HIS, which is vital for optimizing its use and enhancing healthcare quality in Palestine.

## Materials and methods

### Study design, setting, and population

This study employed a quantitative cross-sectional design to examine the factors influencing the acceptance of the Avicenna Health Information System (HIS) among healthcare providers at the Palestine Medical Complex (PMC) in Ramallah, West Bank. The PMC, comprising four governmental hospitals, was selected due to its comprehensive services and diverse workforce, making it an ideal setting for this study. The study population included all healthcare providers working at the PMC, ensuring a broad representation of various professional roles and demographics. The PMC has been using the Avicenna HIS since 2010 to improve healthcare delivery, making it a relevant environment for assessing HIS acceptance.

### Study sample

A systematic random sampling technique was used to select the study participants from a population of 900 healthcare providers working at the PMC, according to 2022 data from the Ministry of Health (MOH). Inclusion criteria required that participants were employed before May 2022to ensure their experience with Avicenna., officially registered with the MOH, and willing to participate in the study. Using the Raosoft formula with a 95% confidence level, 0.5 response distribution, and a 0.050 margin of error, the sample size was calculated to be 274. To account for a 10% attrition rate, the target sample size was adjusted to 300 providers.

The process of systematic random sampling involved assigning numbers to all 900 healthcare providers and selecting every third individual from a randomly chosen starting point. This method ensured an unbiased and representative inclusion of various professional roles, such as doctors, nurses, midwives, pharmacists, laboratory technicians, managers, physiotherapists, and radiology technicians.

Each selected healthcare provider was personally contacted initially by phone and asked to participate in the study. The research team met with each selected participant face-to-face, provided a brief verbal explanation of the research and its significance, and obtained written informed consent before administering the self-administered questionnaire through the iPad. The research team waited on-site for each participant to complete the questionnaire to ensure immediate response and minimize non-response bias.

### Data collection tool

The study utilized a self-administered questionnaire adapted from a structured tool designed by Handayani et al. (1) and Pai and Huang (16), based on the Technology Acceptance Model (TAM), to evaluate healthcare providers' acceptance of the HIS.

To evaluate hospital information system acceptance among healthcare providers. Minor modifications were made to align the questionnaire with the standards of the Palestine Medical Complex, ensuring its relevance and applicability to the specific context of this study.

The content validity of the questionnaire was assessed by a panel of four multidisciplinary experts who reviewed the questionnaire for relevance, comprehensiveness, and clarity. Based on their feedback and suggestions, modifications were made to ensure that the questionnaire comprehensively covered all relevant aspects of HIS acceptance. A pilot study was conducted with 10% of the sample size from the PMC, and these participants were excluded from the final study. The pilot aimed to test the clarity of the questionnaire, assess the appropriateness of the study instruments, and evaluate the clarity of meanings and scales. The reliability of the questionnaire was evaluated using the internal consistency test, specifically by calculating Cronbach's alpha. The reliability coefficient was found to be 0.86, indicating a high degree of reliability.

### Translation process

To ensure that the questionnaire was culturally appropriate and accurately reflected the original English content in Arabic, a back-translation process based on WHO guidelines for back translation was conducted (16). The English version of the questionnaire was first translated into Arabic by two independent field experts fluent in both languages and knowledgeable in health information systems. A back-translation of the Arabic version was then performed, converting it back into English. This back-translated version was compared with the original English questionnaire to identify any discrepancies or potential misinterpretations. This process ensured that the questions retained the same meaning and context across both languages, thereby enhancing the validity and cross-cultural applicability of the questionnaire.

### Ethical considerations and administrative procedures

This study was conducted in accordance with ethical standards and was approved by the Institutional Review Board (IRB) at An-Najah National University under reference number Mas. July, 2022/2. Official permissions were obtained from the Ministry of Health and the administration of the Palestine Medical Complex (PMC) to conduct the research. Written informed consent was obtained from all participants after providing them with a comprehensive explanation of the study's objectives, procedures, and their rights as participants.

### Statistical analysis

The data analysis was performed using IBM SPSS version 25.0, employing both descriptive and inferential statistics. Descriptive statistics, such as frequencies, percentages, means, and standard deviations, were used to summarize demographic data and scores on various scales measuring HIS acceptance. Reliability analysis was conducted using Cronbach's alpha to assess the internal consistency of the questionnaire. Inferential statistics included independent sample t-tests and one-way ANOVA to examine differences in mean scores across demographic groups. Prior to analysis, data were checked for completeness and normality to ensure the validity of the statistical methods applied.

## Results

### Sample characteristics

A total of 300 healthcare providers at the Palestine Medical Complex (PMC) participated in the study, resulting in a 100% response rate. The sample comprised 178 males (59.3%) and 122 females (40.7%). The majority of participants were aged between 20 and 39 years (270 participants, 90%), with 159 (53.0%) in the 20–29 age category and 111 (37.0%) in the 30–39 age category. Most participants held a bachelor's degree (250 participants, 83.3%), while 25 (8.3%) had a diploma, and 25 (8.3%) had a master's degree. Nurses constituted the largest professional group (153 participants, 51.0%), followed by doctors (72 participants, 24.0%). Other roles included midwives, pharmacists, laboratory technicians, managers, physiotherapists, and radiology technicians. In terms of experience at PMC, 129 participants (43.0%) had 1–5 years of experience, 112 (37.3%) had 6–10 years, 52 (17.3%) had 11–15 years, and 7 (2.3%) had 16 years or more. (See Table 1 for details).

**Table 1:** Demographical characteristic of the study participants (*n* = 300).

Variable	Values	Frequency	Percentage
Gender	Male	178	59.3%
	Female	122	40.7%
Age category	20–29 years old	159	53.0%
	30–39 years old	111	37.0%
	40–49 years old	26	8.7%
	50 years and older	4	1.3%
Educational level	Diploma degree	25	8.3%
	Bachelor's degree	250	83.3%
	Master's degree	25	8.3%
Job title	Nurse	153	51.0%
	Midwife	12	4.0%
	Doctor	72	24.0%
	Pharmacist	9	3.0%
	Laboratory technician	12	4.0%
	Manager	19	6.3%
	Physiotherapist	6	2.0%
	Radiology technician	17	5.7%
	1–5 years	129	43.0%
	6–10 years	112	37.3%
	11–15 years	52	17.3%
	16 years or more	7	2.3%

### Usefulness and ease of use

The Health Information System (HIS) was rated highly by participants in terms of perceived usefulness and ease of use, both scoring a mean of 4.511 (SD = 0.295). This indicates a strong agreement among healthcare providers regarding the benefits and usability of the HIS in their daily practice. Male participants rated the usefulness and ease of use significantly higher (mean = 4.56) compared to female participants (mean = 4.43), with a *t*-test result of *t* = 3.75 and a p-value of <0.001. Age also influenced these scores, with younger participants (20–29 years old) rating the HIS highest (mean = 4.60), showing a significant difference across age groups (ANOVA *F* = 6.42, *p* < 0.001). Experience level further impacted these perceptions, as those with 1–5 years of experience rated the system more positively (mean = 4.60) compared to those with longer tenures (ANOVA *F* = 8.15, *p* < 0.001) (See Table 3 for details).

### Technological factors

Technological factors, including system quality and information quality, were also positively rated, with a mean score of 4.004 (SD = 0.228). This reflects the perceived reliability and functionality of the HIS in supporting healthcare tasks, as shown in Table 2. The ratings for technological factors showed no significant differences across gender, age, educational level, or job title (all *p*-values >0.05). However, there was a slight variation based on experience, although it was not statistically significant (ANOVA *F* = 0.34, *p* = 0.764) (See Table 3 for details).

**Table 2:** Descriptive statistics of the overall scores of the scale's domains.

Scale domain	Mean	St. Dev	Minimum	Maximum
Usefulness	4.511	0.295	3.38	5.00
Ease of use	4.511	0.295	3.38	5.00
Human factors	3.510	0.197	3.00	4.00
Technological factors	4.004	0.228	3.13	4.50
Organizational factors	2.858	0.304	2.00	4.00
Acceptance	4.218	0.387	3.00	5.00
Overall	3.744	0.132	3.29	4.15

**Table 3:** Difference in mean scores of usefulness, ease of use, human factors, technological factors, and organizational factors affecting HIS acceptance according to participants’ demographic factors.

Demographic factor	Values	Usefulness & ease of use			Human factors			Technological factors			Organizational factors			Acceptance factors		
		Mean	*p*-value	*t*/*F*^a^	Mean	*p*-value	*t*/*F*	Mean	*p*-value	*t*/*F*	Mean	*p*-value	*t*/*F*	Mean	*p*-value	*t*/*F*
Gender	Male	4.56	<0.001	*t* = 3.75	3.52	0.061	*t* = 1.88	3.99	0.289	*t* = −1.07	2.83	0.068	*t* = −1.84	4.22	0.789	*t* = 0.27
	Female	4.43			3.48			4.02			2.89			4.21		
Age category	20–29 years old	4.60	<0.001	*F* = 0.12	3.51	0.936	*F* = 0.12	3.99	0.423	*F* = 0.94	2.79	<0.001	*F* = 5.61	4.35	<0.001	*F* = 8.72
	30–39 years old	4.43			3.50			4.03			2.95			4.16		
	40–49 years old	4.38			3.51			3.98			2.81			3.82		
	50 years and older	4.25			3.51			3.90			2.96			3.19		
Educational level	Diploma degree	4.45	0.558	*F* = 0.59	3.56	0.079	*F* = 2.58	4.04	0.612	*F* = 0.51	2.97	0.076	*F* = 2.75	4.15	0.544	*F* = 0.61
	Bachelor's degree	4.52			3.51			4.00			2.84			4.22		
	Master's degree	4.52			3.43			3.98			2.92			4.27		
Job title	Nurse	4.47	0.128	*F* = 1.78	3.51	0.839	*F* = 0.46	4.01	0.733	*F* = 0.68	2.87	0.477	*F* = 0.94	4.23	0.596	*F* = 0.75
	Midwife	4.52			3.51			4.02			2.86			4.23		
	Doctor	4.57			3.48			3.97			2.83			4.20		
	Pharmacist	4.67			3.58			3.91			2.88			4.39		
	Laboratory technician	4.65			3.55			4.00			2.94			4.13		
	Manager	4.53			3.53			3.98			2.75			4.21		
	Physiotherapist	4.48			3.48			4.04			2.708			3.96		
	Radiology technician	4.46			3.51			4.06			2.92			4.26		
Experience years in Palestine Medical Complex (PMC)	1–5 years	4.60	<0.001	*F* = 8.15	3.54	0.047	*F* = 3.10	4.01	0.764	*F* = 0.34	2.82	0.244	*F* = 1.37	4.36	<0.001	*F* = 9.81
	6–10 years	4.54			3.48			4.00			2.90			4.20		
	11–15 years	4.29			3.49			3.98			2.86			3.96		
	16 years or more	4.00			3.38			4.07			2.76			3.96		

*P* < 0.05.

^a^ *t* = *t*-test/*F* = ANOVA.

### Human factors

Human factors, encompassing aspects such as user confidence and perceived ease in learning the system, received a mean score of 3.510 (SD = 0.197), as detailed in Table 2. Differences in human factor ratings were not significant across gender (*t*-test *t* = 1.88, *p* = 0.061) or age categories (ANOVA *F* = 0.12, *p* = 0.936). However, educational level showed a trend towards significance (ANOVA *F* = 2.58, *p* = 0.079), with diploma holders rating human factors slightly higher than other groups. Experience was a significant factor, with those having 1–5 years of experience rating human factors more positively (mean = 3.54, ANOVA *F* = 3.10, *p* = 0.047) compared to those with longer tenure (See Table 3 for details).

### Organizational factors

Organizational factors, which include management support, training, and resources, received the lowest mean score of 2.858 (SD = 0.304), as shown in Table 2. This suggests a need for improvement in organizational support to enhance HIS acceptance. A significant difference in organizational factors was observed across age groups (ANOVA *F* = 5.61, *p* < 0.001), indicating older participants perceived organizational support differently. Experience levels did not show significant differences in organizational factor ratings (ANOVA *F* = 1.37, *p* = 0.244) (See Table 3).

### Acceptance factors

Overall acceptance of the HIS among healthcare providers was moderately high, with a mean score of 4.218 (SD = 0.387), as outlined in Table 2. This indicates a general willingness among healthcare providers to use the HIS despite challenges related to organizational support and system integration. Acceptance scores varied significantly with experience, with those having 1–5 years of experience reporting higher acceptance (mean = 4.36, ANOVA *F* = 9.81, *p* < 0.001) (See Table 3). These findings indicate that demographic factors such as gender, age, and experience can influence healthcare providers' perceptions of HIS usefulness and ease of use, highlighting the need for targeted strategies to enhance HIS acceptance across different groups.

## Discussion

This study aimed to identify the factors influencing the acceptance of Health Information Systems (HIS) among healthcare providers in Palestine. The findings emphasize the significant role of technological factors, particularly system quality and information quality, in shaping user acceptance. A reliable HIS enhances healthcare delivery efficiency and effectiveness by providing accessible and comprehensive information (18), aligning with global healthcare standards that prioritize the delivery of high-quality medical care (19).

Ensuring continuity of care is a critical factor in HIS acceptance. The adoption of HIS promotes a patient-centered approach, facilitating collaboration among healthcare professionals and improving the quality of medical services (1, 18). Effective information sharing strengthens trust within the healthcare team and supports smoother transitions between healthcare facilities, ultimately enhancing patient outcomes (13).

Technological factors, such as system functionality and data quality, significantly impact HIS acceptance. A well-functioning HIS that meets these standards is essential for user satisfaction and engagement (4). However, challenges like system integration and response times remain, particularly during peak usage periods. The increasing number of users has strained the system, highlighting the need for continuous upgrades and support (20).

Chong et al. (21) further indicate that HIS acceptance is influenced by multiple predictors, including effort expectancy, technology perceptions, and performance expectancy. Addressing both direct and indirect factors, such as user attitudes and training, is essential for optimizing technology use in healthcare. Tetik et al. (22) emphasize that tailored training programs significantly improve perceived usefulness and ease of use, suggesting that comprehensive training is crucial for HIS adoption.

Human factors also play a significant role in HIS acceptance. Younger and less experienced healthcare providers find HIS more useful and easier to use, which aligns with the Technology Acceptance Model (TAM).Enhancing digital literacy and providing adequate training can improve HIS acceptance across different provider groups (1). Additionally, Abu-Snieneh et al. (23) highlight the influence of external factors like attachment to technology and social media could affect HIS acceptance and usage among healthcare providers. Addressing these factors through comprehensive training and user-centered design can enhance system adoption and performance.

Organizational factors, including management support and user involvement, are vital for HIS adoption but often lack adequate attention. Improving these areas through effective management practices and comprehensive training is necessary for successful HIS implementation (6, 24, 25). In addition to these factors, fostering a health-promoting environment and addressing psychological well-being are important for HIS adoption. Health-promoting behaviors and supportive interventions can enhance HIS acceptance and improve overall healthcare quality (25–27).

## Data Availability

The datasets presented in this study can be found in online repositories. The names of the repository/repositories and accession number(s) can be found in the article/Supplementary Material.
